# Pituitary neuroendocrine tumor: A neuropsychological comparison with intra‐axial tumor

**DOI:** 10.1002/acn3.52022

**Published:** 2024-02-22

**Authors:** Hanlu Tang, Yehong Fang, Zhixu Bie, Heyuan Jia, Bo Wang, Zhijun Yang, Ruolin Yang, Zhixian Gao, Xingchao Wang, Pinan Liu

**Affiliations:** ^1^ Department of Neurosurgery Beijing Tiantan Hospital, Capital Medical University Beijing China; ^2^ Clinical College of Neurology, Neurosurgery and Neurorehabilitation Tianjin Medical University Tianjin 300070 China; ^3^ School of Instrumentation and Optoelectronic Engineering Beihang University Beijing 100191 China; ^4^ School of Psychological and Cognitive Sciences and Beijing Key Laboratory of Behavior and Mental Health Peking University Beijing 100871 China; ^5^ IDG/McGovern Institute for Brain Research Peking University Beijing 100871 China; ^6^ Peking‐Tsinghua Center for Life Sciences Peking University Beijing 100871 China; ^7^ Key Laboratory of Machine Perception, Ministry of Education Peking University Beijing 100871 China; ^8^ Department of Neural Reconstruction Beijing Neurosurgery Institute, Capital Medical University Beijing China

## Abstract

**Objective:**

Despite pituitary neuroendocrine tumor (PitNET) being extra‐axial tumors without direct damage to brain tissue, patients with PitNET exhibit neuropsychological impairments. However, it remains unclear whether there are neuropsychological differences between PitNET and intra‐axial tumors that directly destroy the brain parenchyma. This prospective study aims to clarify this distinction to inform decision‐making for intracranial tumors of diverse origins.

**Methods:**

A total of 146 patients with PitNET, 74 patients with glioma representing intra‐axial tumors, and 52 age‐, sex‐, and education‐matched healthy controls were recruited. All patients received standard treatment and postoperative rehabilitation. Clinical data were meticulously collected, and neuropsychological tests were administered to all participants both before and 3 months after surgery.

**Results:**

Both PitNET and glioma patients experience the dual burden of cognitive and affective deficits. However, the feature of these deficits differs substantially. In PitNET patients, the deficits are relatively mild and focal, whereas in glioma patients, they are severe and extensive. Specifically, PitNET patients exhibit deficits in memory, anxiety, and negative affect. In contrast, glioma patients display deficits in executive function, attention, anxiety, positive/negative affect, and empathy. Notably, except for persistent memory deficits, the majority of neuropsychological scores declines in PitNET patients are restorable and can reach improvement within a short period after standard surgical therapy and perioperative management. Conversely, glioma patients not only fail to show improvements but also demonstrate worsening in terms of general cognition and memory postoperatively.

**Interpretation:**

As an extra‐axial tumor, PitNET may exhibit distinctive cognitive and affective functioning compared to intra‐axial tumors, highlighting the need for specific treatment approaches for PitNET patients.

## Introduction

Brain tumors cause not only neurological symptoms but also cognitive^1^, ^2^ and affective impairments^3^, ^4^, ^5^ that reduce patient quality of life.^6^ In the clinic, we focus most on neuropsychological changes associated with intra‐axial tumors, which directly destroy brain parenchyma^7^ and result in obvious neuropsychological deficits. Among these, gliomas, the most prevalent primary brain tumors, have been extensively investigated. Compared with intra‐axial tumors, neuropsychological deficits resulting from extra‐axial brain tumors have historically received much less attention from researchers. For example, pituitary neuroendocrine tumors (PitNETs), the second most common primary brain tumors, originate in the sellar region.^8^, ^9^ These tumors compress surrounding brain tissue^10^, ^11^ and exhibit abnormal hormone secretion.

Prior researches have reported cognitive impairments in PitNET patients,^11^, ^12^ encompassing domains such as attention,^13^ memory,^11^, ^13^, ^14^ and executive function,^15^ alongside affective disturbances like depression^16^ and anxiety.^12^, ^17^ However, it remains unclear the distinction between neuropsychological status in PitNET patients and those with intra‐axial tumors, as well as the effect of the surgical intervention on their neuropsychological improvement, which limits selecting clinical management approaches for neuropsychological status in these brain tumors from different locations.

The present study aims to elucidate the characteristics of neuropsychological dysfunction in individuals diagnosed with PitNET, juxtaposing these findings with intra‐axial tumors, specifically glioma, which serves as a representative subset. Glioma, being not only the most common primary brain tumor but also the most prevalent intra‐axial tumor with a substantial population, has been extensively studied regarding the neuropsychological status of patients, yielding abundant related data. We employed a comprehensive neuropsychological test battery, considering the gold standard,^18^ to probe cognitive and affective domains. Testing was performed twice, that is, pre‐ and postoperatively, shedding light on the impact of surgery on cognitive and affective functions. Additionally, we conducted an in‐depth analysis to discern any associations between various clinical factors and neuropsychological impairments.

## Methods

### Study design

**Figure 1:**
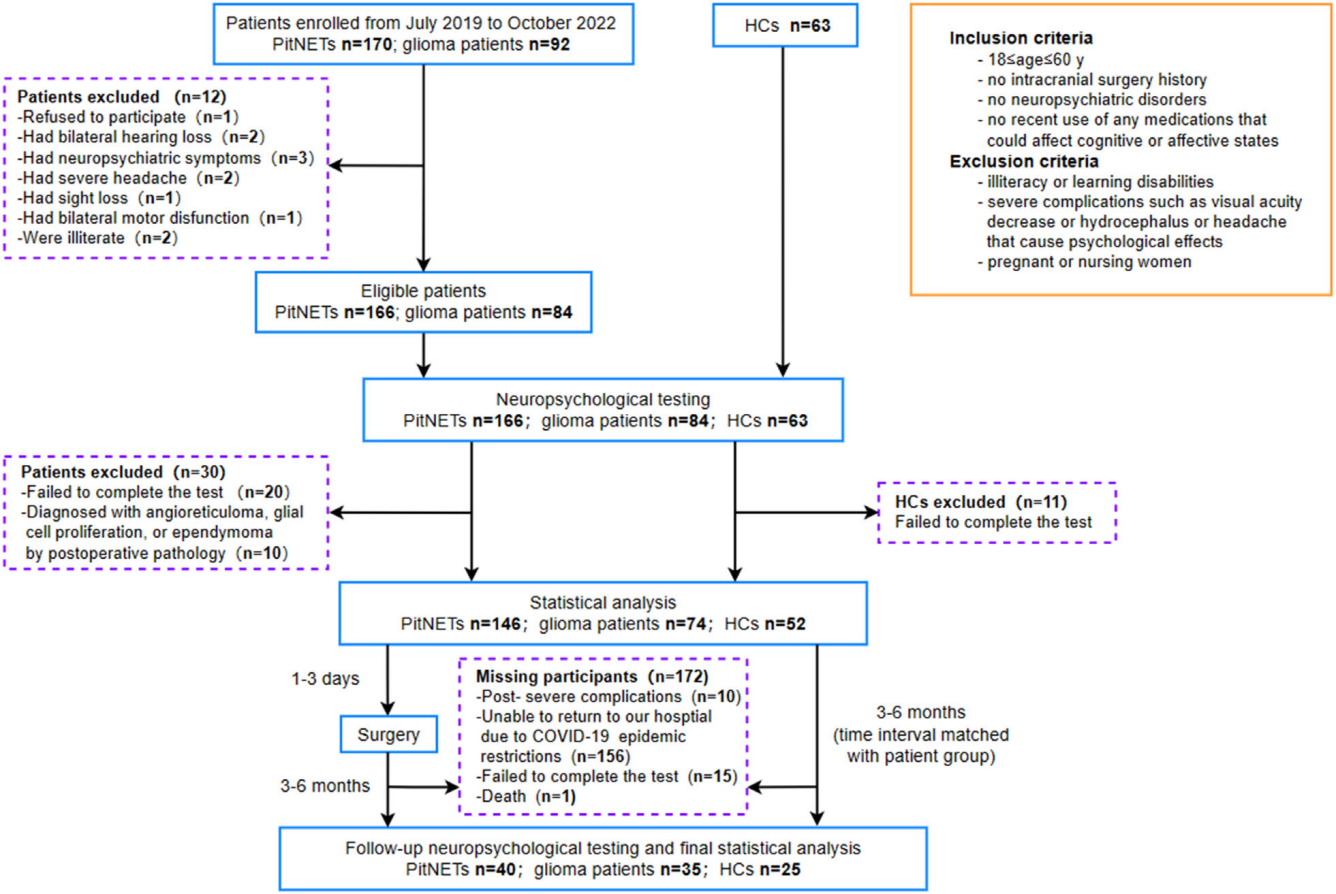
**Flow diagram showing the participant enrollment and screening process, inclusion/exclusion criteria, study design, and testing timeline.** HCs, healthy controls; PitNETs, pituitary neuroendocrine tumors.

We recruited individuals diagnosed with PitNET or glioma who sought treatment at Beijing Tiantan Hospital between July 2019 and October 2022. Healthy controls (HCs) were recruited from the local community. The inclusion/exclusion criteria, enrollment process, excluded patients, and testing timeline are detailed in Figure 1. Ultimately, our analysis included 146 patients with PitNET, 74 patients with gliomas, and 52 HCs. This prospective study received approval from the Medical Ethics Committee of our hospital, and all participants gave informed consent.

### Evaluation of clinical indicators

All patients received standard treatment and perioperative management. Postoperative endocrine reexamination was periodically monitored for PitNET patients, and hormone replacement therapy was conducted for pituitary insufficiency. We collected general demographic information, including sex, age, education level, and dominant hand. Additionally, we gathered clinical data, such as disease course, symptoms, radiological findings, endocrinal results, surgical approach, and pathological reports. Clinical symptoms encompassed both endocrine and neurological manifestations. Radiological data were obtained via T1‐weighted gadolinium‐enhanced and FLAIR sequences using a 3.0 Tesla scanner (TRIO; Siemens, Erlangen, Germany).^19^, ^20^ Endocrinological data for pituitary hormones were collected by analyzing fasting morning blood samples. All surgical procedures and pathological diagnoses were performed by experienced neurosurgeons and pathologists. Follow‐up assessments were recommended at 3, 6, and 12 months, and yearly thereafter.^9^

### Neuropsychological assessment

The neuropsychological assessment covered both cognitive (including general cognitive status, executive function, memory, and attention) and affective (including anxiety, depression, positive/negative affect, and empathy) domains. We administered a test battery comprising 10 scales: the mini–mental state examination (MMSE),^21^ the Montreal Cognitive Assessment (MoCA),^22^ the Frontal Assessment Battery (FAB),^23^ the Wechsler Adult Intelligence Scale–Fourth Edition Digit Span Test (DST; including the Digit Span‐Forward [DST‐f], Digit Span‐Backward [DST‐b], and Digit Span‐Sort [DST‐s] tests),^24^ the Trail Making Test‐part A (TMT‐A) and Trail Making Test‐part B (TMT‐B),^25^, ^26^ the Attentional Control Scale (ACS),^27^ the Hamilton Anxiety Scale (HAMA),^28^ the Beck Depression Inventory (BDI),^29^ the Positive and Negative Affect Scale (PANAS) including the positive subscale (PANASp) and negative subscale (PANASn),^30^ and the Interpersonal Reactivity Index (IRI).^31^, ^32^ All tests were conducted by a skilled psychologist in a quiet room, with an average duration of approximately 60 min. All patients underwent two assessments that took place 1–3 days before surgery and during a follow‐up visit after surgery. The HCs also completed two assessments at equivalent time points.

### Statistical analysis

We conducted the statistical analysis using SPSS software (ver. 22.0; IBM Corp., Armonk, NY, USA). We calculated the z‐standardized score for each subject: first calculate the mean (μ) and standard deviation (s) of the scores for each domain in the first test of HCs, and then calculate z‐standardized score = (original score − μ)/s. Continuous variables are presented as the mean ± standard deviation. Data with an approximately normal distribution and satisfying the assumption of homogeneity of variance were subjected to an analysis of variance, followed by Bonferroni's post hoc test. Heterogeneous data were analyzed using Welch's analysis of variance, and pairs of data were compared using Tamhane's T2 test. We used Student's *t*‐tests to compare two sets of data. For continuous variables with a non‐Gaussian distribution, we used the Kruskal–Wallis H test to compare more than two groups, and the Mann–Whitney *U* test to compare two groups. Categorical variables, expressed as the number of cases (percentage), were subjected to the chi‐squared test or Fisher's exact test. In addition, after matching for parameters such as age and education level, we conducted Pearson and Spearman correlation analyses (for homogeneous and heterogeneous data, respectively) of the neuropsychological scores and clinical indicators (including disease course, tumor size, and suprasellar extension of PitNET). For individual‐level analyses, we conducted z‐tests to compare the mean standardized z‐scores for each cognitive and affective scale of preoperative PitNET patients to the HCs. A mean z‐score ± 1 SD of HCs was considered within the normal range, while a z‐score greater or below this range indicated abnormality.^11^ The level of statistical significance was set at *p* < 0.05.

## Results

### Demographic and clinical data

Table 1 provides an overview of the demographic and clinical characteristics of the study population. No significant differences were observed among PitNET patients, glioma patients, and HCs with respect to sex, age, education level, or dominant hand. The mean tumor size of PitNET was significantly smaller than that of glioma (6.25 ± 7.03 vs. 35.85 ± 33.11 cm^3^, *p* < 0.001).

**Table 1:** Demographic and clinical characteristics of the participants.

Values	PitNET (*n* = 146)	Glioma (*n* = 74)	HCs (*n* = 52)	*p*‐value
Sex (M/F)	71:75 (48.63%:51.37%)	40:34 (54.05%:45.95%)	19:33 (36.54%:63.46%)	0.146
Age (y)	39.26 ± 9.45	40.64 ± 10.94	36.54 ± 10.08	0.201
Education (y)	13.82 ± 3.54	13.58 ± 3.11	12.75 ± 3.21	0.193
Dominant hand (R/L)	138:8 (94.52%:5.48%)	66:8 (89.19%:10.81%)	49:3 (94.23%:5.77%)	0.317
Disease course (mos)	18.79 ± 27.43	10.83 ± 23.38	–	**<0.001**
Symptom				
Visual field defect/hypopsia	52 (35.62%)	4 (5.41%)	–	**<0.001**
Amenorrhea/decreased libido	51 (34.93%)	–	–	–
Acromegaly	45 (30.82%)	–	–	–
Cushing syndrome	6 (4.11%)	–	–	–
Headache	48 (32.88%)	13 (17.57%)	–	**0.015**
Dizzy	18 (12.33%)	17 (22.97%)	–	**0.044**
Epilepsy	–	34 (45.95%)	–	–
Nausea/vomit	–	10 (13.51%)	–	–
Language disorder	–	4 (5.41%)	–	–
Motor/sense dysfunction	–	13 (17.57%)	–	–
Functional/nonfunctional	61:85 (41.78%:58.22%)	–	–	–
Location				
Sellar region	146 (100%)	–	–	–
Frontal lobe	–	40 (54.05%)	–	–
Insular	–	17 (22.97%)	–	–
Parietal lobe	–	11 (14.86%)	–	–
Temporal lobe	–	6 (8.11%)	–	–
Occipital lobe	–	0	–	–
Laterality (R/L)	–	35:39 (47.30%:52.70%)	–	–
Dominant hemisphere tumor	–	37 (50.00%)	–	–
Tumor size (cm^3^)	6.25 ± 7.03	35.85 ± 33.11	–	**<0.001**
Suprasellar extension	96 (65.75%)	–	–	–
Suprasellar extension (mm)	11.47 ± 5.68	–	–	–
Surgical approach				
Endonasal transsphenoidal	140 (95.89%)	–	–	–
Transcranial	6 (4.11%)	74 (100%)	–	–
Pathology				
Gonadotroph adenoma	52 (35.62%)	–	–	–
Somatotroph adenoma	39 (26.71%)	–	–	–
Corticotroph adenoma	27 (18.49%)	–	–	–
Lactotroph adenoma	16 (10.96%)	–	–	–
Null cell adenoma	11 (7.53%)	–	–	–
Thyrotroph adenoma	1 (0.68%)	–	–	–
WHO I	–	6 (8.11%)	–	–
WHO II	–	39 (52.70%)	–	–
WHO III	–	20 (27.03%)	–	–
WHO IV	–	9 (12.16%)	–	–

Boldface type indicates statistically significant differences.

“–”, not applicable; HCs, healthy controls; L, left; PitNET, pituitary neuroendocrine tumor; R, right.

### Preoperative comparison of PitNET patients, glioma patients, and HCs

Table 2 presents the preoperative cognitive and affective scores for the three groups in our study. Patients with PitNET exhibited a notable decline in memory test performance compared to HCs (DST, *p* = 0.020; DST‐s, *p* = 0.006), although no deficits were observed in general cognitive function, executive function, or attention. Individual‐level analysis revealed memory deficits in 42 (28.76%) PitNET patients. Patients with glioma showed poorer executive function (FAB, *p* = 0.001) and attention (ACS, *p* < 0.001; attention focusing subscale, *p* < 0.001; attention diversion subscale, *p* < 0.001) than HCs. No deficits in general cognitive function or memory were observed in the glioma group. Patients with PitNET showed better executive function (FAB, *p* = 0.002) and attention (ACS, *p* < 0.001; attention focusing subscale, *p* < 0.001; attention diversion subscale, *p* < 0.001) than those in the glioma group.

**Table 2:** Cognitive and affective functioning of the participants.

Values	PitNET (*n* = 146)	Glioma (*n* = 74)	HCs (*n* = 52)	*F* score	*p*‐value
General cognitive function					
MMSE	−0.00 ± 1.12	−0.40 ± 1.42	0.00 ± 1.00	–	0.111
MoCA	−0.27 ± 0.97	−0.11 ± 1.09	0.00 ± 1.00	–	0.148
Executive function					
TMT‐B	0.22 ± 1.11	0.65 ± 1.52	0.00 ± 1.00	–	0.050^b^
FAB	−0.24 ± 1.35	−1.24 ± 2.35	0.00 ± 1.00	–	**<0.001** ^b^ ^c^
Memory					
DST	−0.43 ± 0.91	−0.22 ± 1.14	0.00 ± 1.00	4.013	**0.019** ^a^
DST‐f	−0.27 ± 0.87	−0.11 ± 1.02	0.00 ± 1.00	–	0.080
DST‐b	−0.36 ± 0.98	−0.25 ± 1.17	0.00 ± 1.00	–	0.121
DST‐s	−0.38 ± 0.83	−0.14 ± 0.98	0.00 ± 1.00	–	**0.005** ^a^
Attention					
TMT‐A	0.16 ± 0.98	0.24 ± 1.17	0.00 ± 1.00	–	0.459
ACS	0.01 ± 1.29	−1.48 ± 0.87	0.00 ± 1.00	63.044	**<0.001** ^b^ ^c^
Attention focusing	−0.12 ± 1.07	−1.62 ± 1.02	0.00 ± 1.00	–	**<0.001** ^b^ ^c^
Attention diversion	0.13 ± 1.28	−0.87 ± 0.83	0.00 ± 1.00	27.381	**<0.001** ^b^ ^c^
Anxiety					
HAMA	0.97 ± 1.54	1.06 ± 1.50	0.00 ± 1.00	–	**<0.001** ^a^ ^b^
Mental	0.57 ± 1.34	0.71 ± 1.38	0.00 ± 1.00	–	**0.010** ^a^ ^b^
Physical	1.32 ± 1.89	1.24 ± 1.75	0.00 ± 1.00	–	**<0.001** ^a^ ^b^
Depression					
BDI	0.18 ± 1.13	0.23 ± 1.08	0.00 ± 1.00	–	0.531
Affection					
PANAS	0.07 ± 0.95	−0.01 ± 0.96	0.00 ± 1.00	0.252	0.778
PANASp	−0.36 ± 1.02	−0.47 ± 0.97	0.00 ± 1.00	–	**0.021** ^b^
PANASn	0.61 ± 1.24	0.63 ± 1.33	0.00 ± 1.00	–	**0.003** ^a^ ^b^
Empathy					
IRI	0.04 ± 1.09	−0.63 ± 0.90	0.00 ± 1.00	11.342	**<0.001** ^b^ ^c^
Perspective taking	0.13 ± 1.13	−0.08 ± 1.06	0.00 ± 1.00	–	0.226
Fantasy	0.17 ± 1.06	−0.23 ± 0.83	0.00 ± 1.00	4.966	**0.008** ^c^
Empathy concern	0.05 ± 1.03	−1.06 ± 0.67	0.00 ± 1.00	–	**<0.001** ^b^ ^c^
Personal distress	−0.24 ± 1.29	−0.24 ± 1.36	0.00 ± 1.00	–	0.469

Boldface type indicates statistically significant differences.

“–”, not applicable; ACS, Attentional Control Scale; BDI, Beck Depression Inventory; DST: Digit Span Test; DST‐b, Wechsler Adult Intelligence Scale—Fourth Edition Digit Span‐Backward; DST‐f, Wechsler Adult Intelligence Scale—Fourth Edition Digit Span‐Forward; DST‐s, Wechsler Adult Intelligence Scale—Fourth Edition Digit Span‐Sort; FAB, Frontal Assessment Battery; HAMA, Hamilton Anxiety Scale; HCs, healthy controls; IRI, Interpersonal Reactivity Index; MMSE, mini‐mental state examination; MoCA, Montreal Cognitive Assessment; PANAS, Positive and Negative Affect Scale; PANASn, Positive and Negative Affect Scale‐negative affect; PANASp, Positive and Negative Affect Scale‐positive affect; PitNET, pituitary neuroendocrine tumor; TMT‐A, Trail‐Making Test part A; TMT‐B, Trial‐Making Test‐part B.

^a^ Significant differences between PitNET patients and HCs (*p* < 0.05).

^b^ Significant differences between glioma patients and HCs (*p* < 0.05).

^c^ Significant differences between PitNET and glioma patients (*p* < 0.05).

Turning to affective domains, the PitNET group showed more anxiety (HAMA, *p* < 0.001; mental subscale, *p* = 0.037; physical subscale, *p* < 0.001) and negative affect (PANASn, *p* = 0.003) than the HCs, although no deficits were observed concerning depression or empathy. Individual‐level analysis showed anxiety in 87 (59.59%) PitNET patients, along with negative affect in 52 (35.61%) PitNET patients. On the other hand, patients with gliomas also exhibited more anxiety (HAMA, *p* < 0.001; mental subscale, *p* = 0.009; physical subscale, *p* < 0.001) and negative affect (PANASn, *p* = 0.014) than HCs. Moreover, there was a noticeable reduction in positive affect (PANASp, *p* = 0.019) and empathy (IRI, *p* = 0.003, empathic concern subscale, *p* < 0.001) within the glioma group in comparison to the HCs. Nevertheless, depression was not observed in the glioma group. Additionally, it is worth noting that patients with PitNET showed a higher capacity for empathy compared to the glioma group (IRI, *p* < 0.001, fantasy subscale, *p* = 0.006, empathic concern subscale, *p* < 0.001).

### Preoperative versus postoperative results

The cognitive and affective changes before and after surgery in both the PitNET and glioma groups are summarized in Tables 3 and 4, respectively. In comparison to the preoperative data, the PitNET group exhibited noteworthy postoperative enhancements in general cognitive function scores (MMSE, *p* = 0.045; MoCA, *p* = 0.034) and executive function scores (TMT‐B, *p* < 0.001; FAB, *p* = 0.025). Improved scores were also observed on the DST‐b memory test (*p* = 0.045) and the TMT‐A attention test (*p* = 0.001). Additionally, the PitNET group showed reduced levels of anxiety (HAMA, *p* = 0.002; mental subscale, *p* = 0.004; physical subscale, *p* = 0.015) and negative affect (PANASn subscale; *p* = 0.045) following surgery. However, no statistically improved scores were observed in the domains of positive affect, depression, or empathy within the PitNET patient group. Conversely, there were no statistically improved scores detected in either cognitive or affective domains within the glioma group following the surgical intervention.

**Table 3:** Comparison between preoperative and postoperative cognitive and affective functioning in participants with PitNET.

Values	Pre‐operation (*n* = 40)	Post‐operation (*n* = 40)	*t*/*Z*	*p*‐value
General cognitive function				
MMSE	0.06 ± 1.09	0.35 ± 0.60	−2.002	**0.045**
MoCA	−0.29 ± 0.95	0.07 ± 0.98	−2.125	**0.034**
Executive function				
TMT‐B	0.46 ± 1.31	−0.18 ± 1.08	−3.929	**<0.001**
FAB	−0.47 ± 1.82	0.20 ± 0.68	−2.235	**0.025**
Working memory				
DST	−0.45 ± 0.85	−0.26 ± 0.97	−1.721	0.093
DST‐f	−0.42 ± 0.87	−0.33 ± 0.91	−0.697	0.490
DST‐b	−0.32 ± 0.86	−0.08 ± 1.01	−2.006	**0.045**
DST‐s	−0.35 ± 0.84	−0.23 ± 0.91	−0.919	0.358
Attention				
TMT‐A	0.15 ± 0.95	−0.25 ± 0.84	−3.347	**0.001**
ACS	−0.22 ± 1.51	0.01 ± 1.37	−1.261	0.215
Attention focusing	−0.27 ± 1.18	−0.12 ± 1.06	−0.924	0.361
Attention diversion	−0.08 ± 1.49	0.13 ± 1.54	−1.174	0.247
Anxiety				
HAMA	0.97 ± 1.37	0.20 ± 1.50	−3.046	**0.002**
Mental	0.41 ± 1.10	−0.12 ± 1.27	−2.843	**0.004**
Physical	1.61 ± 1.99	0.72 ± 1.71	−2.427	**0.015**
Depression				
BDI	0.30 ± 1.41	−0.09 ± 0.88	−1.467	0.142
Affection				
PANAS	−0.02 ± 0.91	−0.07 ± 1.02	0.317	0.753
PANASp	−0.36 ± 0.97	−0.13 ± 1.06	−1.600	0.118
PANASn	0.46 ± 1.07	0.06 ± 1.10	−2.005	**0.045**
Empathy				
IRI	0.13 ± 1.19	0.17 ± 1.06	−0.249	0.805
Perspective taking	0.13 ± 1.11	0.00 ± 1.11	−0.941	0.347
Fantasy	0.30 ± 1.16	0.44 ± 0.99	−0.673	0.505
Empathy concern	0.28 ± 0.97	0.24 ± 0.75	−0.582	0.561
Personal distress	−0.32 ± 1.50	−0.35 ± 1.47	−0.103	0.918

Boldface type indicates statistically significant differences.

ACS, Attentional Control Scale; BDI, Beck Depression Inventory; DST: Digit Span Test; DST‐b, Wechsler Adult Intelligence Scale—Fourth Edition Digit Span‐Backward; DST‐f, Wechsler Adult Intelligence Scale—Fourth Edition Digit Span‐Forward; DST‐s, Wechsler Adult Intelligence Scale—Fourth Edition Digit Span‐Sort; FAB, Frontal Assessment Battery; HAMA, Hamilton Anxiety Scale; IRI, Interpersonal Reactivity Index; MMSE, mini‐mental state examination; MoCA, Montreal Cognitive Assessment; PANAS, Positive and Negative Affect Scale; PANASn, Positive and Negative Affect Scale‐negative affect; PANASp, Positive And Negative Affect Scale‐positive affect; PitNET, pituitary neuroendocrine tumor; TMT‐A, Trail‐Making Test‐part A; TMT‐B, Trial‐Making Test‐part B.

**Table 4:** Comparison between preoperative and postoperative cognitive and affective functioning in participants with glioma.

Values	Pre‐operation (*n* = 35)	Post‐operation (*n* = 35)	*t*/*Z*	*p* value
General cognitive function				
MMSE	−0.33 ± 1.50	−0.31 ± 1.85	−0.738	0.460
MoCA	−0.13 ± 1.24	−0.34 ± 1.50	−0.431	0.666
Executive function				
TMT‐B	0.87 ± 1.72	0.67 ± 1.54	−0.118	0.906
FAB	−1.69 ± 2.78	−2.01 ± 3.94	−0.525	0.599
Working memory				
DST	−0.38 ± 1.26	−0.30 ± 1.42	−0.361	0.720
DST‐f	−0.24 ± 1.15	−0.26 ± 1.26	−0.021	0.983
DST‐b	−0.38 ± 1.25	−0.15 ± 1.38	−1.178	0.239
DST‐s	−0.28 ± 1.04	−0.31 ± 1.09	−0.218	0.828
Attention				
TMT‐A	0.39 ± 1.45	0.57 ± 1.51	−0.527	0.598
ACS	−1.41 ± 0.77	−1.56 ± 1.03	−0.984	0.325
Attention focusing	−1.49 ± 0.78	−1.32 ± 1.13	−0.056	0.955
Attention diversion	−0.89 ± 0.83	−1.27 ± 0.83	−1.953	0.051
Anxiety				
HAMA	1.00 ± 1.53	0.72 ± 2.00	−0.948	0.343
Mental	0.62 ± 1.44	0.38 ± 1.61	−0.618	0.537
Physical	1.28 ± 1.76	1.03 ± 2.26	−0.866	0.387
Depression				
BDI	0.08 ± 1.09	0.14 ± 1.00	−0.592	0.554
Affection				
PANAS	−0.02 ± 1.00	−0.36 ± 1.08	1.456	0.155
PANASp	−0.47 ± 1.02	−0.70 ± 1.14	−0.762	0.446
PANASn	0.61 ± 1.52	0.39 ± 1.30	−0.814	0.416
Empathy				
IRI	−0.60 ± 0.78	−0.72 ± 1.05	0.647	0.522
Perspective taking	−0.04 ± 1.08	−0.29 ± 1.05	0.967	0.341
Fantasy	−0.08 ± 0.76	−0.34 ± 1.28	1.317	0.197
Empathy concern	−1.23 ± 0.56	−1.17 ± 0.74	−0.458	0.650
Personal distress	−0.18 ± 1.42	−0.03 ± 1.14	−0.569	0.573

ACS, Attentional Control Scale; BDI, Beck Depression Inventory; DST: Digit Span Test; DST‐b, Wechsler Adult Intelligence Scale—Fourth Edition Digit Span‐Backward; DST‐f, Wechsler Adult Intelligence Scale—Fourth Edition Digit Span‐Forward; DST‐s, Wechsler Adult Intelligence Scale—Fourth Edition Digit Span‐Sort; FAB, Frontal Assessment Battery; HAMA, Hamilton Anxiety Scale; IRI, Interpersonal Reactivity Index; MMSE, mini‐mental state examination; MoCA, Montreal Cognitive Assessment; PANAS, Positive and Negative Affect Scale; PANASn, Positive and Negative Affect Scale‐negative affect; PANASp, Positive and Negative Affect Scale‐positive affect; TMT‐A, Trail‐Making Test‐part A; TMT‐B, Trial‐Making Test‐part B.

To mitigate the potential influence of practice effects, HCs underwent a second assessment after the initial evaluation, with the same time interval as that used for the other two groups (Table S1). This additional assessment revealed no statistically significant differences, except for the mental subscale of the HAMA (*p* = 0.015).

### Postoperative comparison of PitNET patients, glioma patients, and HCs

The postoperative cognitive and affective scores of PitNET and glioma patients, along with the results of the second assessment of HCs, are summarized in Table 5. There were no significant differences in the time interval between the two neuropsychological tests among the three groups (*p* = 0.333).

**Table 5:** Demographic, clinical, cognitive, and affective comparison among the participants at follow‐up.

Values	PitNET (*n* = 40)	Glioma (*n* = 35)	HCs (*n* = 25)	*F* score	*p* value
Sex (M/F)	20:20 (50.00%:50.00%)	16:19 (45.71%:54.29%)	9:16 (36.00%:64.00%)	–	0.541
Age (y)	38.25 ± 8.70	39.31 ± 11.07	35.36 ± 10.16	–	0.504
Education (y)	12.88 ± 3.30	13.37 ± 2.81	12.76 ± 3.55		0.700
Dominant hand (R/L)	39:1 (97.50%:2.50%)	30:5 (85.71%:14.29%)	24:1 (96.00%:4.00%)		0.140
Time interval (mos)	3.48 ± 1.74	4.94 ± 3.93	3.36 ± 0.64	–	0.333
General cognitive function					
MMSE	0.33 ± 0.60	−0.30 ± 1.90	0.24 ± 0.93	–	0.060
MoCA	0.05 ± 0.98	−0.35 ± 1.53	0.55 ± 0.96	–	**0.014** ^b^
Executive function					
TMT‐B	−0.18 ± 1.08	0.67 ± 1.54	−0.61 ± 0.71	–	**0.003** ^b^
FAB	0.14 ± 0.76	−1.75 ± 3.91	0.24 ± 0.63	–	**<0.001** ^b^ ^c^
Memory					
DST	−0.28 ± 0.98	−0.22 ± 1.40	0.61 ± 1.01	5.182	**0.007** ^a^ ^b^
DST‐f	−0.34 ± 0.92	−0.26 ± 1.24	0.17 ± 0.95	–	0.149
DST‐b	−0.08 ± 1.02	−0.05 ± 1.35	0.54 ± 1.09	–	0.065
DST‐s	−0.26 ± 0.90	−0.23 ± 1.06	0.74 ± 1.05	9.154	**<0.001** ^a^ ^b^
Attention					
TMT‐A	−0.27 ± 0.85	0.46 ± 1.47	−0.33 ± 0.95	–	**0.022** ^b^
ACS	−0.00 ± 1.39	−1.48 ± 0.95	0.19 ± 1.18	20.826	**<0.001** ^b^ ^c^
Attention focusing	−0.11 ± 1.07	−1.24 ± 1.10	0.04 ± 0.92	–	**<0.001** ^b^ ^c^
Attention diversion	0.10 ± 1.41	−1.21 ± 0.77	0.26 ± 1.44	–	**<0.001** ^b^ ^c^
Anxiety					
HAMA	0.19 ± 1.51	0.67 ± 2.05	−0.54 ± 0.68	–	**0.033** ^b^
Mental	−0.15 ± 1.27	0.38 ± 1.66	−0.71 ± 0.52	–	**0.008** ^b^
Physical	0.76 ± 1.72	0.94 ± 2.28	0.03 ± 1.05	–	0.133
Depression					
BDI	−0.08 ± 0.89	0.16 ± 1.03	−0.35 ± 0.51	–	0.197
Affection					
PANAS	−0.08 ± 1.03	−0.28 ± 1.05	−0.64 ± 1.00	1.925	0.151
PANASp	−0.13 ± 1.07	−0.65 ± 1.14	−0.53 ± 0.87	3.027	0.053
PANASn	0.05 ± 1.11	0.45 ± 1.31	−0.30 ± 0.72	–	0.248
Empathy					
IRI	0.13 ± 1.04	−0.69 ± 1.00	−0.30 ± 1.08	6.545	**0.002** ^c^
Perspective taking	−0.03 ± 1.10	−0.22 ± 0.96	−0.14 ± 0.83	–	0.793
Fantasy	0.42 ± 1.00	−0.37 ± 1.25	0.08 ± 1.23	4.127	**0.019** ^c^
Empathy concern	0.23 ± 0.75	−1.19 ± 0.75	1.19 ± 2.50	–	**<0.001** ^b^ ^c^
Personal distress	−0.40 ± 1.45	0.03 ± 1.13	0.04 ± 1.04	–	0.530

Boldface type indicates statistically significant differences.

“–”, not applicable; ACS, Attentional Control Scale; BDI, Beck Depression Inventory; DST: Digit Span Test; DST‐b, Wechsler Adult Intelligence Scale—Fourth Edition Digit Span‐Backward; DST‐f, Wechsler Adult Intelligence Scale—Fourth Edition Digit Span‐Forward; DST‐s, Wechsler Adult Intelligence Scale—Fourth Edition Digit Span‐Sort; FAB, Frontal Assessment Battery; HAMA, Hamilton Anxiety Scale; HCs, healthy controls; IRI, Interpersonal Reactivity Index; MMSE, mini‐mental state examination; MoCA, Montreal Cognitive Assessment; PANAS, Positive and Negative Affect Scale; PANASn, Positive and Negative Affect Scale‐negative affect; PANASp, Positive and Negative Affect Scale‐positive affect; PitNET, pituitary neuroendocrine tumor; TMT‐A, Trail‐Making Test‐part A; TMT‐B, Trial‐Making Test‐part B.

^a^ Significant differences between PitNET patients and HCs (*p* < 0.05).

^b^ Significant differences between glioma patients and HCs (*p* < 0.05).

^c^ Significant differences between PitNET and glioma patients (*p* < 0.05).

In consonance with the presurgical evaluation, the PitNET group continued to exhibit diminished postoperative memory performance in comparison to the HCs (DST, *p* = 0.016; DST‐s, *p* = 0.001). Diverging from the presurgical assessment, the glioma group not only persisted in displaying poorer executive function (TMT‐B, *p* = 0.003; FAB, *p* = 0.002) and attention (TMT‐A, *p* < 0.001; attention focusing subscale, *p* < 0.001; attention diversion subscale, *p* < 0.001) than the HCs after surgery but also manifested new deficits in general cognitive function (MoCA, *p* = 0.014) and memory (DST, *p* = 0.014; DST‐s, *p* < 0.001). Furthermore, mirroring the presurgical evaluation, the PitNET group continued to demonstrate superior executive function (FAB, *p* = 0.002) and attention (ACS, *p* < 0.001; attention focusing subscale, *p* < 0.001; attention diversion subscale, *p* < 0.001) relative to the glioma group postoperatively.

Regarding the affective domains, the PitNET group did not exhibit any postoperative deficit in any affective test. Conversely, the glioma group continued to display increased anxiety levels (HAMA, *p* = 0.032; mental subscale, *p* = 0.006) and lower levels of empathy compared to the HCs (empathic concern subscale, *p* < 0.001). Additionally, the PitNET group maintained a higher level of empathy (IRI, *p* = 0.001; fantasy subscale, *p* = 0.015; empathic concern subscale, *p* < 0.001) than the glioma group postoperatively.

### Correlation analysis

**Figure 2:**
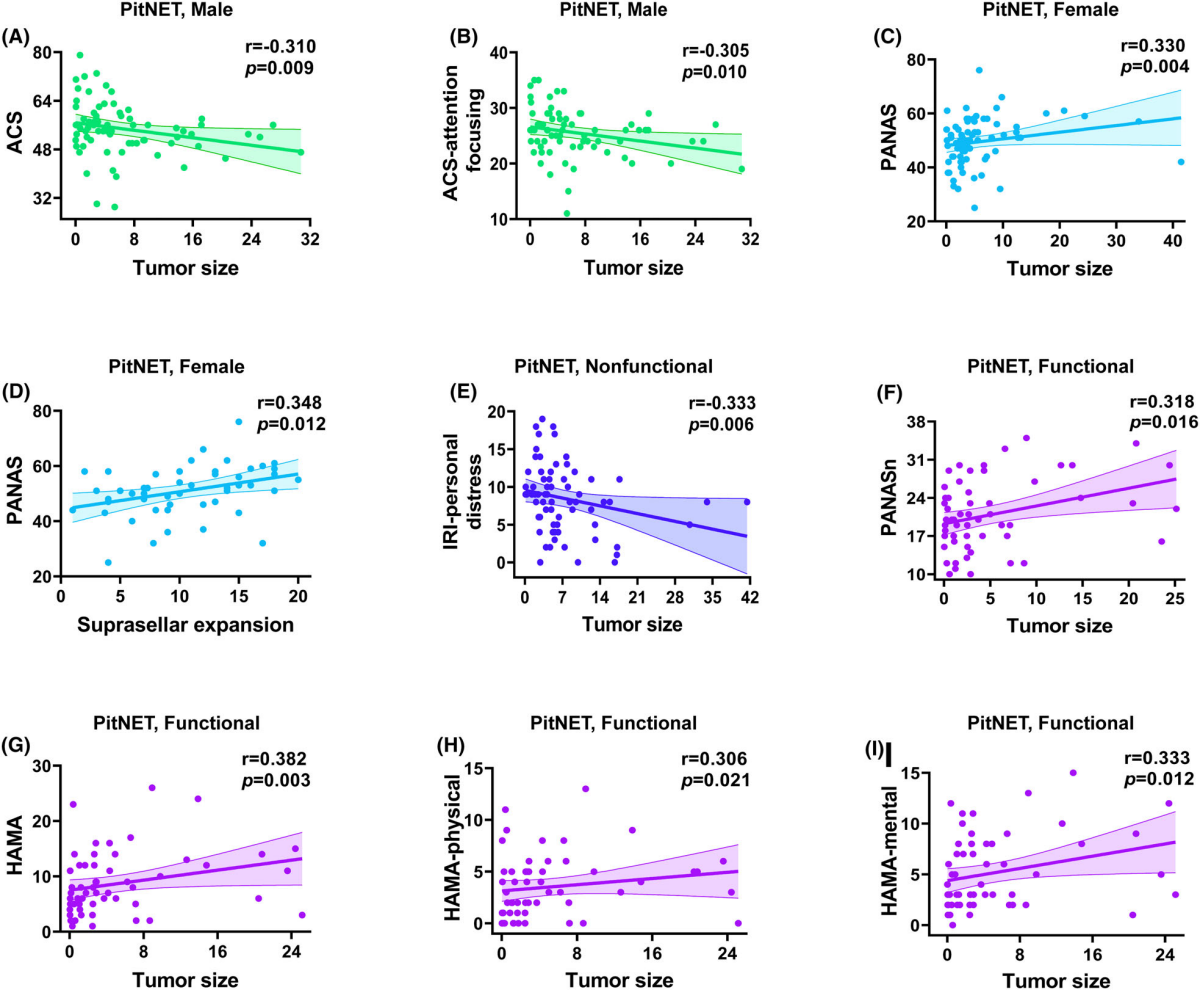
**Correlations of clinical variables with cognitive and affective deficits in PitNET patients (A–I)**. Only correlations significant at *p* < 0.05 are shown. PitNET, pituitary neuroendocrine tumors; ACS, Attentional Control Scale; HAMA, Hamilton Anxiety Scale; IRI, Interpersonal Reactivity Index; PANAS, Positive and Negative Affect Scale; PANASn, Positive and Negative Affect Scale‐negative affect; PANASp, Positive and Negative Affect Scale‐positive affect.

**Figure 3:**
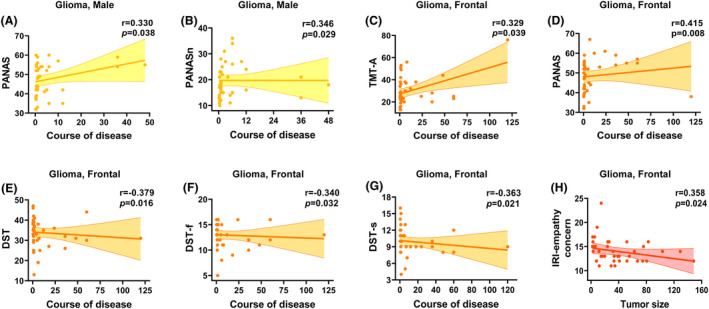
**Correlations of clinical variables with cognitive and affective deficits in glioma patients (A–H).** Only correlations significant at *p* < 0.05 are shown. DST, Wechsler Adult Intelligence Scale–Fourth Edition Digit Span Test; DST‐f, Wechsler Adult Intelligence Scale–Fourth Edition Digit Span‐Forward; DST‐s, Wechsler Adult Intelligence Scale–Fourth Edition Digit Span‐Sort; IRI, Interpersonal Reactivity Index; PANAS, Positive and Negative Affect Scale; PANASn, Positive and Negative Affect Scale‐negative affect; PANASp, Positive and Negative Affect Scale‐positive affect; TMT‐A, Trail Making Test‐part A.

Correlations between clinical variables and cognitive/affective functions in the PitNET and glioma groups are presented in Figures 2 and 3. In the PitNET group, some cognitive and affective functions were associated with tumor size rather than disease course. Specifically, larger tumor size was correlated with worse attention, more anxiety, abnormal affect, and low empathy. In contrast, in the glioma group, some cognitive and affective functions were associated with disease course rather than tumor size. Specifically, a longer disease course was correlated with worse memory, poor executive function, abnormal affect, and low empathy.

## Discussion

In the current study, we investigated the neuropsychological difference between PitNET and glioma by enrolling 272 participants (including 146 PitNET patients, 74 glioma patients, and 52 HCs). Our findings revealed that neuropsychological impairments in PitNET share both similarities and distinctions with those in glioma patients. Moreover, the response to surgical intervention for neuropsychological impairments differed significantly between PitNET and glioma patients. To the best of our knowledge, this is the first study to comprehensively evaluate the neuropsychological status of PitNET patients using intra‐axial tumor patients as a disease control.

### Mild and focal cognitive and affective impairments in PitNET compared to glioma

Although both PitNET and glioma patients experience the dual burden of cognitive and affective deficits, the nature of these deficits differs substantially. In PitNET patients, the deficits are relatively mild and focal compared to HCs, whereas in glioma patients, they are severe and extensive compared to HCs. Specifically, PitNET patients exhibited no deficits in most cognitive domains (including general cognitive function, executive function, and attention) except for the memory domain. In contrast, patients with gliomas showed deficits in executive function and attention but not memory. In terms of affective domains, PitNET patients exhibited anxiety (both mental and physical anxiety) and negative affect, with no deficits in terms of depression, positive affect, or empathy. Conversely, patients with gliomas not only showed anxiety (mental and physical) and negative affect as PitNET patients but also exhibited little positive affect and low empathy.

It is worth noting that our study included various types of PitNET as well as different grades and locations of glioma. Previous studies reported both commonalities and distinctions in neuropsychological characteristics among these subtypes. For example, patients with prolactin adenoma presented deficits in verbal/nonverbal memory and attention.^13^ Patients with somatotroph adenoma showed deficits in executive function,^6^, ^15^ attention,^14^ and depression.^12^, ^16^ Patients with corticotroph adenoma exhibited deficits in memory, executive function, and depression.^33^ Patients with nonfunctional adenoma displayed deficits in memory and attention.^11^ Regarding glioma, previous studies reported memory and executive deficits in patients with frontal glioma,^34^, ^35^ impaired empathy ability in those with insular glioma,^36^ verbal working memory deficits in those with low‐grade glioma,^37^ and anxiety and depression in those with high‐grade glioma.^38^ Although each subtype has unique feature, our study mainly focuses on delineating the neuropsychological distinctions between PitNET and glioma patients. The aim is to facilitate the initial screening and assessment of neuropsychological status rather than individualized assessment. Consequently, we include all PitNET and glioma subtypes collectively for analysis and comparison, which may explain some inconsistencies in our results compared to previous findings. Additionally, the methods used to assess neuropsychological domains varied among studies. Each scale has a specific focus, which may also account for some inconsistent results.

### Restorable cognitive and affective impairments in PitNET but not glioma patients after surgery

The difference in neuropsychological resilience between PitNET and glioma patients is striking. PitNET patients showed statistically significant improvements of presurgery anxiety and negative affect at the 3 months follow‐up. Additionally, surgical intervention also ameliorated the initially low preoperative scores of many cognitive scales in PitNET patients, bringing them closer to the levels observed in healthy controls. This observation aligns with prior studies on PitNET patients, which also reported improvements in some cognitive domains following surgery.^39^, ^40^ This rapid short‐term recovery underscores the positive effect of standard surgical therapy and perioperative management on neuropsychological functioning in PitNET patients. Thus, an aggressive and appropriate treatment strategy is warranted for PitNET patients because the impairments were reversible. Conversely, glioma patients not only failed to show any improvement but also exhibited worse general cognition and memory postoperatively. Surgery plays a limited role of improving neuropsychological status due to irreversible impairments. We should explore alternative treatment approaches to ameliorate neuropsychological status.

### Persistent memory deficits in PitNET patients

Additionally, it is noteworthy that no statistical improvement was observed in memory deficits in PitNET patients. Butterbrod et al.^11^ also reported continuous postoperative memory deficits in PitNET patients at the 3 months follow‐up. These findings suggest that memory deficits may be irreversible during the short‐term period compared to other cognitive deficits in PitNET patients. The neural mechanisms underlying this memory deficit remain unclear. We infer that it may attribute to the PitNET tumor compressing surrounding frontal lobe or the hippocampal structure within the temporal lobe, which being associated with memory functioning.^41^ We also do not preclude the possibility that the memory deficits may reach an improvement at a long‐term follow‐up observation, spanning 1 or 2 years. In a word, memory deficits require more extended care and long‐term management scheme.

### Factors impacting cognitive and affective functioning in PitNET patients

The microstructural changes in the brain are different between PitNET and glioma. PitNET has a relatively mild and noninvasive impact on brain tissue. PitNET tends to indirectly influence brain function via the abnormal secretion of hormones (excessive or insufficient)^15^, ^42^, ^43^ or compressing the surrounding brain tissue.^44^ In contrast, glioma tends to result in the destructive infiltration of the cortical and subcortical structures that are crucial for cognition.^7^ These different microstructural changes may explain the milder cognitive and affective impairments in PitNET patients compared to glioma patients.

Regarding the rapid postsurgical improvements observed in PitNET patients, it is plausible to speculate that tumor resection reduces secretions from active tumor cells and relieves the compression on surrounding brain tissue. Conversely, due to the growth of glioma merging with normal brain tissue, the inevitable brain tissue resection following the surgical intervention results in the limited improvement of neuropsychological impairments and may even lead to further neuropsychological deficits. Another possible factor may be the fact that PitNET is mainly operated through endonasal transsphenoidal approach while glioma is operated transcranially. Nevertheless, a previous study found that no statistically neuropsychological difference was observed between endonasal transsphenoidal and transcranial approach in PitNET patients.^39^ Therefore, we infer that the surgical approach may be not the primary cause of postoperative neuropsychological differences.

In addition, cognitive and affective deficits in brain tumor patients are related to various clinical factors, such as tumor size, tumor growth rates,^1^ abnormal neurotransmitter activity,^45^ headaches,^46^ and epilepsy.^47^ In our study, we observed that cognitive and affective impairments were primarily associated with larger tumor size in PitNET patients, rather than the duration of the disease course. Conversely, in glioma patients, cognitive and affective impairments were more strongly linked to the duration of the disease course rather than tumor size. Our findings indicate that it is crucial to provide additional attention and care to neuropsychological problems in PitNET patients with larger tumors and glioma patients with longer disease course.

### Limitations

Several limitations of this study should be acknowledged. First, because of COVID‐19‐pandemic‐related restrictions on mobility, some patients were lost to follow‐up. Although this limited the data that could be collected, we believe that the obtained data are adequate to assess postsurgical cognitive and affective status. Second, the average follow‐up duration is relatively short (3 months). A long‐term follow‐up study is therefore needed in the future. Despite these limitations, our results provide valuable insights into the cognitive and affective functioning of PitNET patients.

## Conclusion

Despite PitNET being an extra‐axial tumor, PitNET patients experience both cognitive and affective impairments. These impairments are distinctive from those in patients with glioma. PitNET patients display mild and focal neuropsychological deficits compared with the severe and extensive dysfunction in glioma (intra‐axial tumors). Moreover, cognitive and affective impairments are restorable in PitNET but not in glioma patients. The scores of many neuropsychological scales can obtain significant amelioration in PitNET patients after standard surgical intervention and perioperative management within a short‐term period (3 months) except for memory. Memory deficits may prove challenging to restore during a short period and require long‐term attention and care. Additionally, some cognitive and affective impairments in PitNET patients may be related to larger tumor size, which suggests that it is important to pay additional attention to neuropsychological problems in PitNET patients with larger tumor. Our findings establish a theoretical foundation for more precise neuropsychological therapies for PitNET patients, and guide different neuropsychological management approaches for intra‐ and extra‐axial tumors.

## Author Contributions

We would like to thank all authors who contributed to the study. Conception and design: Pinan Liu, Xingchao Wang, and Hanlu Tang. Methodology: Hanlu Tang and Xingchao Wang. Data acquisition: Hanlu Tang, Yehong Fang, Zhixu Bie, Ruolin Yang, and Heyuan Jia. Writing—original draft preparation: Hanlu Tang and Yehong Fang. Writing review and editing: Pinan Liu, Bo Wang, Zhijun Yang, and Zhixian Gao. Supervision: Pinan Liu and Xingchao Wang. We also thank all participants who took part in the study.

## Conflict of Interest

No authors have any potential conflicts of interest to disclose.

## Supporting information


Table S1.
