# Supplementary material for: Pituitary neuroendocrine tumor: A neuropsychological comparison with intra‐axial tumor
Source: Ann Clin Transl Neurol. 2024 Feb 22;11(4):1021–33. doi: 10.1002/acn3.52022 (PMC11021612; doi:10.1002/acn3.52022)
Supplement: Supplementary file 1 — Table S1. [file ACN3-11-1021-s001.docx]

**Table S1. Comparison of cognitive and affective functioning between the first and the second test of the HCs**

| **Values** |  | | **First (n=25)** | **Second (n=25)** | **t/Z** | ***p* value** |
| --- | --- | --- | --- | --- | --- | --- |
| **General cognitive function** | |  |  |  |  |  |
| MMSE | |  | 0.02±0.88 | 0.23±0.91 | -0.884 | 0.377 |
| MoCA |  | | 0.39±1.01 | 0.60±0.97 | -0.516 | 0.606 |
| **Executive function** |  | |  |  |  |  |
| TMT-B |  | | -0.44±0.68 | -0.53±0.80 | 0.498 | 0.618 |
| FAB |  | | 0.34±0.45 | 0.25±0.62 | -0.577 | 0.564 |
| **Working memory** |  | |  |  |  |  |
| DST |  | | 0.26±0.93 | 0.58±1.01 | -1.111 | 0.278 |
| DST-f |  | | 0.18±0.89 | 0.15±0.94 | -0.164 | 0.870 |
| DST-b |  | | 0.14±1.03 | 0.50±1.09 | -1.107 | 0.268 |
| DST-s |  | | 0.30±0.92 | 0.72±1.03 | -1.398 | 0.175 |
| **Attention** |  | |  |  |  |  |
| TMT-A |  | | -0.29±0.88 | -0.34±0.93 | -0.309 | 0.757 |
| ACS |  | | 0.01±0.91 | 0.17±1.16 | -0.609 | 0.548 |
| Attention focusing |  | | 0.10±1.23 | -0.18±0.81 | 0.630 | 0.540 |
| Attention diversion |  | | -0.16±1.05 | 0.30±1.48 | -1.537 | 0.148 |
| **Anxiety** |  | |  |  |  |  |
| HAMA |  | | -0.07±1.05 | -0.31±1.32 | -1.368 | 0.171 |
| Mental |  | | -0.01±1.06 | -0.58±0.56 | -1.968 | **0.049** |
| Physical |  | | 0.03±1.13 | 0.21±1.24 | -0.045 | 0.964 |
| **Depression** |  | |  |  |  |  |
| BDI |  | | 0.00±0.98 | -0.26±0.66 | -1.146 | 0.252 |
| **Affection** |  | |  |  |  |  |
| PANAS |  | | -0.27±1.04 | -0.58±1.03 | 1.133 | 0.268 |
| PANASp |  | | -0.20±0.89 | -0.54±0.85 | 1.342 | 0.192 |
| PANASn |  | | -0.15±0.89 | -0.19±0.90 | -0.054 | 0.957 |
| **Empathy** |  | |  |  |  |  |
| IRI |  | | -0.32±0.97 | -0.28±1.07 | -0.158 | 0.876 |
| Perspective taking |  | | -0.44±0.90 | -0.16±0.61 | -0.954 | 0.358 |
| Fantasy |  | | -0.21±1.24 | 0.12±1.41 | -0.646 | 0.529 |
| Empathy concern |  | | -0.17±1.25 | 1.38±2.61 | -1.706 | 0.088 |
| Personal distress |  | | -0.14±0.89 | 0.01±1.06 | -0.587 | 0.563 |

ACS, attentional control scale; BDI, Beck depression inventory; DST: digit span test; DST-b, Wechsler adult intelligence scale fourth edition digit span-backward; DST-f, Wechsler adult intelligence scale fourth edition digit span-forward; DST-s, Wechsler adult intelligence scale fourth edition digit span-sort; FAB, frontal assessment battery; HAMA, Hamilton Anxiety Scale; HCs, healthy controls; IRI, interpersonal reactivity index; MMSE, mini-mental state examination; MoCA, Montreal cognitive assessment; PANAS, positive and negative affect scale; PANASn, positive and negative affect scale negative affect; PANASp, positive and negative affect scale positive affect; TMT-A, trail-making test part A; TMT-B, trial-making test part B.

Boldface type indicates statistically significant differences.
